# Characterization of inorganic phosphate transport in the triple-negative breast cancer cell line, MDA-MB-231

**DOI:** 10.1371/journal.pone.0191270

**Published:** 2018-02-07

**Authors:** Thais Russo-Abrahão, Marco Antônio Lacerda-Abreu, Tainá Gomes, Daniela Cosentino-Gomes, Ayra Diandra Carvalho-de-Araújo, Mariana Figueiredo Rodrigues, Ana Carolina Leal de Oliveira, Franklin David Rumjanek, Robson de Queiroz Monteiro, José Roberto Meyer-Fernandes

**Affiliations:** 1 Instituto de Bioquímica Médica Leopoldo De Meis, Centro de Ciências da Saúde, Universidade Federal do Rio de Janeiro, Rio de Janeiro, RJ, Brazil; 2 Instituto Nacional de Ciência e Tecnologia em Biologia Estrutural e Bioimagem, Rio de Janeiro, RJ, Brazil; Meharry Medical College, UNITED STATES

## Abstract

**Background:**

Recent studies demonstrate that interstitial inorganic phosphate is significantly elevated in the breast cancer microenvironment as compared to normal tissue. In addition it has been shown that breast cancer cells express high levels of the NaP_i_-IIb carrier (SLC34A2), suggesting that this carrier may play a role in breast cancer progression. However, the biochemical behavior of inorganic phosphate (P_i_) transporter in this cancer type remains elusive.

**Methods:**

In this work, we characterize the kinetic parameters of Pi transport in the aggressive human breast cancer cell line, MDA-MB-231, and correlated P_i_ transport with cell migration and adhesion.

**Results:**

We determined the influence of sodium concentration, pH, metabolic inhibitors, as well as the affinity for inorganic phosphate in P_i_ transport. We observed that the inorganic phosphate is dependent on sodium transport (K_0,5_ value = 21.98 mM for NaCl). Furthermore, the transport is modulated by different pH values and increasing concentrations of P_i_, following the Michaelis-Menten kinetics (K_0,5_ = 0.08 mM P_i_). PFA, monensin, furosemide and ouabain inhibited P_i_ transport, cell migration and adhesion.

**Conclusions:**

Taken together, these results showed that the uptake of P_i_ in MDA-MB-231 cells is modulated by sodium and by regulatory mechanisms of intracellular sodium gradient.

General Significance: Pi transport might be regarded as a potential target for therapy against tumor progression.

## Introduction

Phosphorus is an essential element found in all forms of life. In mammals, it is obtained from the diet in the anionic form of Pi (H_2_PO^-4^ or HPO^-4^) through absorption in the small intestine [1]. Inorganic phosphate (Pi) plays a critical role in several cellular processes such as energy metabolism, either in the form of ATP by the energy transfer mechanism, or in its free form as substrates for intermediates of metabolic pathways. As a constituent of ATP, Pi also participates in the mechanisms of phosphorylation and dephosphorylation of intermediates of cellular signaling events. Pi is a fundamental component of phospholipids and nucleotides of DNA and RNA [2]. To satisfy the phosphorus requirements of a healthy mammal, the extracellular Pi is maintained under a relatively narrow range of concentration, between 0.8 and 1.5 mM [1]. Under physiological conditions, Pi is an anionic molecule and, therefore, its diffusion through the membrane lipid bilayer is hindered, requiring thus a protein transport system for translocation from the extracellular to the intracellular medium [3]. In mammals, several inorganic phosphate transporters have been described and can be classified into three different protein families denoted NaPi-I, NaPi-II and NaPi-III. NaPi-I (NPTI) is predominantly expressed in the border membrane of the proximal tubular brush, and functions as an intrinsic Pi transport modulator, [4]. NaPi-II (SLC34) is a sodium-dependent phosphate transporter that carries the phosphate in its monovalent form. It is further classified in the following subfamilies: NaPi-IIa (SLC34A1), NaPi-IIb (SLC34A2), and NaPi-IIc (SLC34A3); NaPi-IIb is found in breast cells [1,5,6,7,8]. The type III, NaPi-III (SLC20) transporters family, comprises proteins responsible for a sodium-dependent phosphate transport in its monovalent form. NaPi-III is divided in two subfamilies: PiT-1 (SLC20A1) and PiT-2 (SLC20A2) [1,4,5,6].

Genome–wide analysis strongly suggests that mutations affect both branches of the regulatory pathways that contribute, simultaneously or not, towards tumorigenesis, namely oncogenes and tumor suppressor genes. These may determine the gain of function of genes dictating growth control and cell survival and inactivate genes that in normal cells promote apoptosis [9]. Given the high degree of interactivity of the network involving cellular signaling pathways and the accumulating evidence assigning to metabolic enzymes the dual role of transcription factors [10], it is not surprising that the cell proliferation and invasiveness typical of cancer cells are intimately connected to metabolic reprogramming and hence a distinct bioenergetic phenotype [11]. The “growth rate hypothesis” was described in cancer cells and it was demonstrated that tumors have elevated phosphorus demands associated with protein synthesis and accelerated proliferation [12]. According to this theory, high phosphorus is required for the rapid growth of tumor, thus having a role in tumorigenesis and tumor progression. Recently, it was observed that the Pi concentration in the tumor microenvironment of breast tumors is significantly elevated as compared to normal mammary glands. In addition, authors documented increased Pi concentrations in highly metastatic tumor xenografts as compared to non-metastatic tumors [13].

There are three important receptors involved with classification of breast cancer: the receptors for the estrogen (ER) and progesterone (PR) hormones or epidermal growth factor receptors (HER-2) [14,15]. According to the classification based on the expression patterns of receptors in breast cancer, there are four molecular subtypes were found: luminal A, luminal B, HER2 and triple-negative overexpression [16]. The MDA-MB-231 cell line (classified in the triple-negative molecular subtype) is a cellular model with a high metastatic capacity and, therefore, is considered a more aggressive strain compared to MCF-7 (ER+, PR+ and HER is classified as a tumorigenic, but non-metastatic cell line) [17,18].

MDA-MB-231 cells exhibit increased migratory capacity upon increasing Pi concentrations in the growth medium. There are two solute carrier families of Pi transport in mammals: SLC20 and SLC34 and both protein families transport Pi using the electrochemical gradient for Na^+^ [19]. It has been previously described that NaPi-IIb (SLC34A2) is up-regulated in ovarian carcinomas and benign tumors compared to normal ovary tissues [20]. Another study showed that the upregulated expression of SLC34A2 in hepatocellular carcinoma cell lines, and the knockdown of this Pi transporter decrease cell proliferation, migration and invasion as well as the epithelial–mesenchymal transition [21]. In lung cancer cells, SLC34A2 was also necessary for proliferation and tumorigenesis [22]. Overexpression of the NaPi-IIb transporter has been described in breast cancer tumors as opposed to normal tissues and has been proposed as a novel diagnostic marker and a therapeutic target [23]. Thus, the aim of this study was to characterize the Pi transport kinetics in the MDA-MB-231 breast cancer cell line by investigating whether such biochemical features may suggest interference tactics using inhibitors that could impact upon migration and invasive capacity.

## Materials and methods

### Materials

Reagents were bought from E. Merck (Darmstadt, Germany) and Sigma Chemical Co. (St. Louis, MO, USA). Radioactive inorganic phosphate (^32^P_i_) used was from Instituto de Pesquisas Energéticas e Nucleares (IPEN). In this work, we used distilled water through a Milli-Q system of resins to prepare all solutions (Millipore Corp., Bedford, MA, USA).

### Cell culture

MDA-MB-231, T47D and MCF-7 cells were grown at 37°C in Iscoves Modified Dulbeco’s Medium (IMDM—LCG Biotechnology, Brazil) supplemented with sodium bicarbonate, 10% of foetal bovine serum (FBS) (Cripion Biotechnology, Brazil), 100 U/mL penicillin and streptomycin (Thermo Fisher, Brazil). 67NR and 4T1 cell lines, which originated from a spontaneous mammary carcinoma arising in a BALB/c mouse [24], were purchased from Karmanos Cancer Institute (Detroit, MI, USA). Cells were maintained in high glucose Dulbecco’s modified Eagle medium (DMEM), supplemented with L-GlutaMax, 10 mM sodium carbonate, Hepes Buffer and 10% FBS and maintained at 37°C in a humidified atmosphere of 5% CO_2_. For the experiments, cells were harvested from the culture medium, washed two times with buffer comprising of 116 mM NaCl, 5.4 mM KCl, 5.5 mM glucose, 0.8 mM MgCl_2_ and 50 mM HEPES (pH 7.2). Hank’s EDTA solution was used to isolated cells from dishes. Cell number was estimated by counting in a Neubauer chamber. The protein concentration was measured with the Bradford methodology [25].

### Pi transport assay in MDA-MB-231

Living MDA-MB-231 cells (10^4^ cells) were incubated at 37°C for 1 hour in a reaction mixture (0.5 mL) containing 116 mM NaCl or choline cloride, 5.4 mM KCl, 5.5 mM glucose, 50 mM HEPES (pH 7.2), 0.8 mM MgCl_2_, 0.1 mM KH_2_PO_4_ and 2.5 μCi/nmol ^32^P_i_ [26]. The reaction was stopped with 0.5 ml of an ice-cold PBS buffer (pH 7.2). Cells were washed with the same cold buffer at 4°C and disrupted with 0.25 mL Hank’s solution (5.37 mM KCl, 0.44 mM KH_2_PO_4_, 136.8 mM NaCl, 0.33 mM NaH_2_PO_4_, 5.03 mM D-glucose, 4.16 mM sodium bicarbonate, 6.35 mM EDTA, pH 7.2) and 0.25 ml 0.1% SDS. These lysed cells with the internalized Pi were moved to a filter paper and, then, to a scintillation liquid. Blank values of uptake were obtained as previously described [26].

It was used 0 to 0.5 mM of Pi to measure the substrate affinity (K_0.5_) and maximum rate (V_max_) of the P_i_ transporter. Bafilomycin A_1_ (100 nM), a vacuolar ATPase inhibitor, and valinomycin (100 μM), the K^+^ ionophore, were also tested. Monensin (100 μM), a Na^+^ ionophore, furosemide (1 mM), a Na^+^-ATPase inhibitor and ouabain (1 mM), a Na^+^,K^+^-inhibitor, were tested. The H^+^,K^+^-ATPase inhibitor, SCH28080 (100 μM) and the P_i_ transport inhibitor, phosphonoformic acid (PFA—5 mM) were also tested. Vehicles: DMSO 1% (bafilomycin A_1_, valinomycin, furosemide and SCH28080), ethanol 1% (monensin), water (ouabain and PFA); controls were performed using these vehicles. At 1% of these vehicles, the values for ^32^P_i_ uptake obtained were the same obtained with water. The viability of MDA-MB-231 was verified according to the manufacturer’s instructions of CellTiter 96® AQueous One Solution Cell Proliferation Assay (MTS) (Promega, EUA).

### Real-time PCR analysis

Total RNA was purified from MDA-MB-231 cells using TRIzol Reagent (Invitrogen, Thermo Fisher Scientific, Massachusetts, USA) as described by manufacturer’s manual. After treatment of RNA with DNase I, a first-strand synthesis kit (Invitrogen) was used to generate full-length cDNA from 1 μg of total RNA. qPCR was carried in StepONE Plus Real Time PCR System (Applied Biosystems, Massachusetts, USA), using a FastStart Master SYBR Green I Kit (from Roche, Mannheim, Germany). The primers for amplification are shown in Table 1. Gene expression data were normalized to an endogenous reference β-actin (ACTB) as previously described [27], and according to the manufacturer’s instructions.

**Table 1:** Primers sequences.

Sequence name	Sense primer (5’– 3’)	Antisense primer (5’– 3’)
NaPi 1	CGTATGTCTTCTCTGGTTCGTTCTG	CGTAAAACTACCAGTGGAAATAGCCC
NaPi-IIa	GTGGCCTCCTTCAACATCCAT	CTGTAAGGAGTCTGGGTGGC
NaPi-IIb	CCCAGCTTATAGTGGAGAGCTTC	GCACCAAATCTTGACAAGACTCTTG
NaPi-IIc	GAATTTCAGAGGGCTTTCAGCG	GAGTCCAACTGCACGATGAGG
ACTB	TGACGTGGACATCCGCAAAG	CTGGAAGGTGGACAGCGAGG

### Migration assay

Tumor cell migration was assayed in a 48-well Boyden chamber (Neuro Probe Microchemotaxis System, Gaithersburg, MD) using 12-μm polycarbonate filters, as previously described [28]. 28 μl of IMDM containing 2.5% FBS was added at the bottom of the chamber as the chemoattractant. At the upper part, 5 x 10^4^ cells/well, pretreated with ouabain, furosemide, monensin or phosphonoformic acid (PFA) for 1h, was added in 50 μL of medium in the absence of FBS. Controls were performed by pretreating the cells, for 1 h, in the specific drug diluents: 1% DMSO or 1% ethanol in 50 μL of medium in the absence of FBS., After migration for 1.5h, filters were removed from the chambers, fixed and stained with Panoptic kit (Interlab, São Paulo, Brazil). Tumor cells that had migrated through the membrane were counted in a LX400 light microscopy under a 100 x objective (Labomed Inc, Los Angeles, CA) on at least five random fields. The results are representative of three independent experiments performed in triplicate.

### Adhesion assay

96-well tissue culture plates were performed pre-coated with 32 μg/mL ECM gel (from mouse sarcoma) diluted in phosphate buffered saline (PBS), 100 μL per well for overnight in 4°C and non-specific binding sites in the wells were blocked with 1 mg/mL BSA diluted in PBS for 2 h at room temperature. Cells grown at 80% confluency in IMDM with 10% FBS were pretreated with the phosphate transport inhibitors for 1h, then the cells were trypsinized, suspended in serum-free medium at the concentration of 2 x 10^4^ cells/mL and applied 100 μL for each well incubated under routine condition as above for 3h. After incubation, non-adherent cells were removed by carefully washing twice with PBS, fixed with 3% paraformaldehyde for 10 min. Cleaned with PBS two times, stained with 100 μL 0.5% crystal violet for 5 min, washed two times and lysed with 100 μL ethanol of 1% acetic acid solution read at A570. Results were expressed 100% as control [29].

### Statistical analysis

All experiments were performed, at least, three times in triplicate. The experiments were represented with values of mean ± SE. We used nonlinear regression analysis of the data to the Michaelis–Menten equation (K_0.5_ and V_max_ values). Significant differences: p<0.05. Statistical analyses were performed using Prism 6.0 software (GraphPad Software, San Diego, USA).

## Results

### Pi transport in different lineages of breast cancer cells

**Fig 1:**
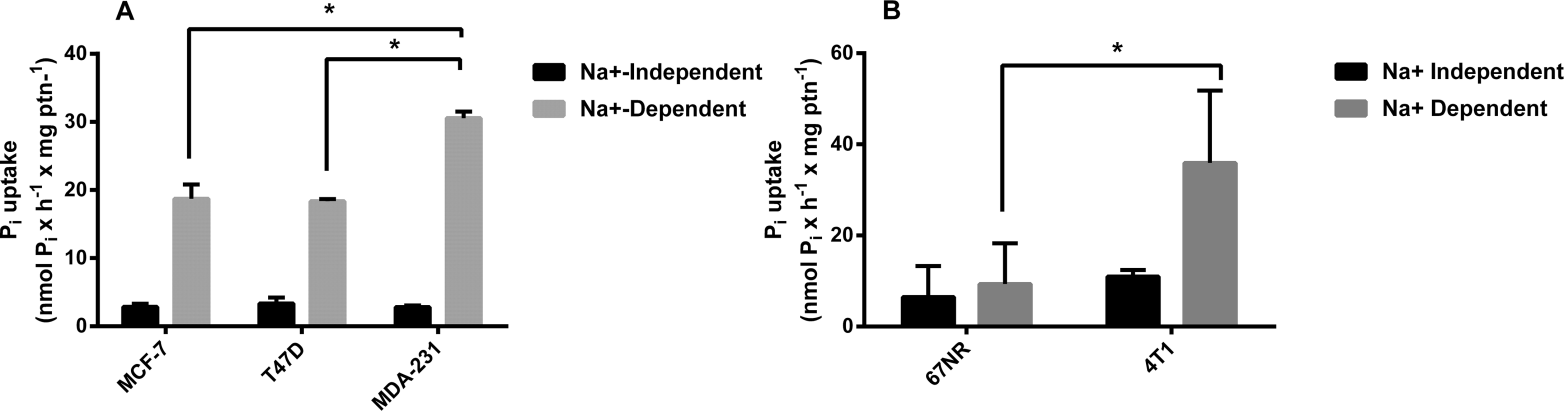
Comparative indices of ^32^Pi influx in breast cancer cell lines. Intact MCF-7, T47-D or MDA-MB-231 cells (5 x 10^4^ cells/mL = 1.45 mg protein/mL) (A) and 67NR and 4T1 (B) were incubated for 1 h at 37°C in a reaction mixture containing 116 mM NaCl or 116 mM choline cloride, 5.5 mM Glucose, 5.4 KCl, 10 mM HEPES, 0.8 mM MgCl_2_, 0.1 mM KH_2_PO_4_ and 2.5 μCi / nmol ^32^Pi. The results are the means ± SE of at least 3 experiments, with different cell suspensions. Asterisks mark significant differences (p≤ 0.05) from MDA-MB-231, as determined by One-Way analysis of variance (ANOVA), using Turkey’s multiple comparisons test.

**Fig 2:**
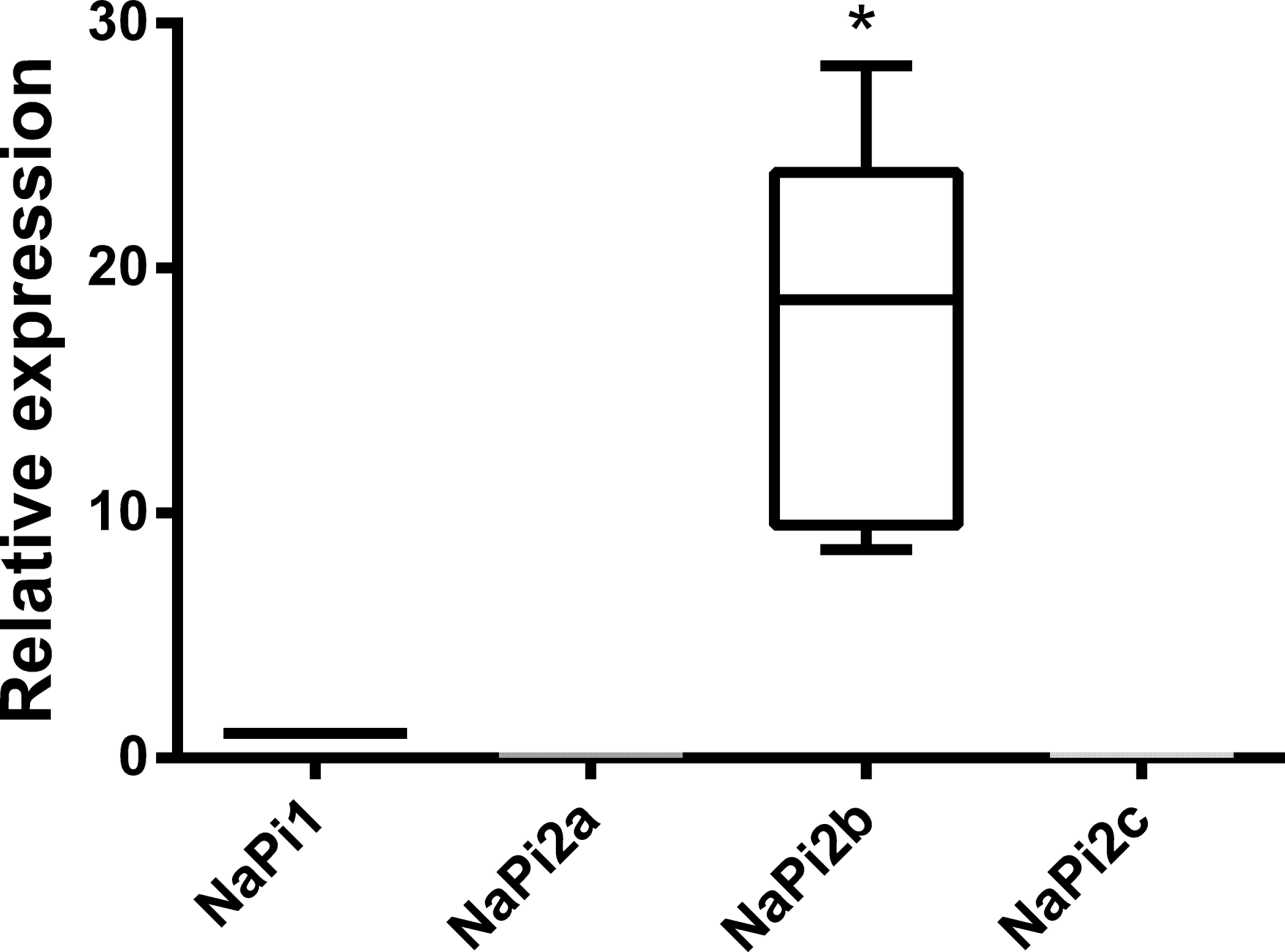
Gene expression analysis of Pi transporters in MDA-MB-231 cells. Total RNA was purified from MDA-MB-231 and, after treatment of RNA with DNase I, a full-length cDNA was generated from RNA. qPCR was carried. Gene expression data were normalized to an endogenous reference β-actin (ACTB). The results are the means ± SE of 7 experiments, with different cell suspensions. Asterisks mark significant differences (p≤ 0.05) from NaPi1, as determined by One-Way analysis of variance (ANOVA), using Turkey’s multiple comparisons test.

In an initial approach, we assayed the Pi transport in three different human breast cancer cell lines. When we compared them, the most aggressive, MDA-MB-231, had the higher P_i_ transport activity (Fig 1A). In addition, we employed two isogenic murine breast cancer cells: 67NR, is a low-aggressive, nonmetastatic, cell line and 4T1, is a highly aggressive, metastatic, cell line [24]. As seen in the human cell lines, 4T1 cells presented higher ^32^P_i_ transport than 67NR (Fig 1B). Based on these initial results, we opted to concentrate our study specifically on the MDA-MB-231-line. We next analyzed the gene expression pattern of Pi transporters. As shown in Fig 2, MDA-MB-231 cells exhibited the highest NaPi-IIb gene expression levels when compared to the other NaPi transporters (Fig 2).

### Pi uptake and kinetic parameters

**Fig 3:**
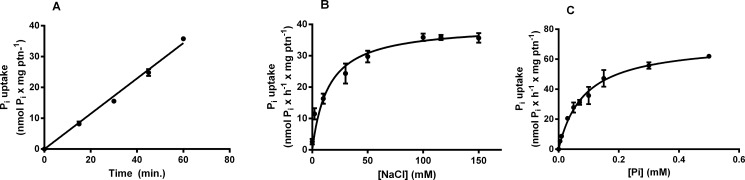
Time course, NaCl dependence and Pi dependence of MDA-MB-231 ^32^Pi influx. Intact cells (5 x 10^4^ cells/mL = 1.45 mg protein/mL) were incubated at 37°C in a reaction mixture containing 116 mM NaCl or 116 mM choline cloride, 5.5 mM Glucose, 5.4 KCl, 10 mM HEPES, 0.8 mM MgCl_2_, 0.1 mM KH_2_PO_4_ and 2.5 μCi/nmol ^32^Pi at various times (A), several NaCl concentrations (0–150 mM) (B) or various concentrations of KH_2_PO_4_ (0–0.5 mM) (C). The results are the means ± SE of at least 3 experiments, with different cell suspensions.

The Pi uptake in MDA-MB-231 was evaluated at different times (0 to 60 minutes) and these cells showed a linear uptake for up to 60 minutes (Fig 3A). We did not measure longer times of incorporation because it could lead to saturation. Then, all experiments were carried out at 60 minutes.

NaP_i_-II (SLC34) is a member of the sodium-dependent P_i_ transporter family and transports the monovalent form of P_i_. Fig 3B shows the influence of NaCl on the Pi transport activity of MDA-MB-231 line. The kinetic parameters of this activity were: K_0.5_ = 14.11 ± 2.838 mM and V_max_ = 39.72 ± 1.26 nmol x h^-1^ x mg protein^-1^.

To evaluate the affinity of the transport for P_i_, we performed the Pi uptake in the P_i_ concentration range of 5–500 μM (Fig 3C). The transporter followed a Michaelis-Menten kinetic and the parameter values were calculated, presenting an apparent K_0.5_ = 84.9 ± 10.4 μM and a V_max_ = 71.67 ± 3 nmol x h^-1^ x mg protein^-1^.

**Fig 4:**
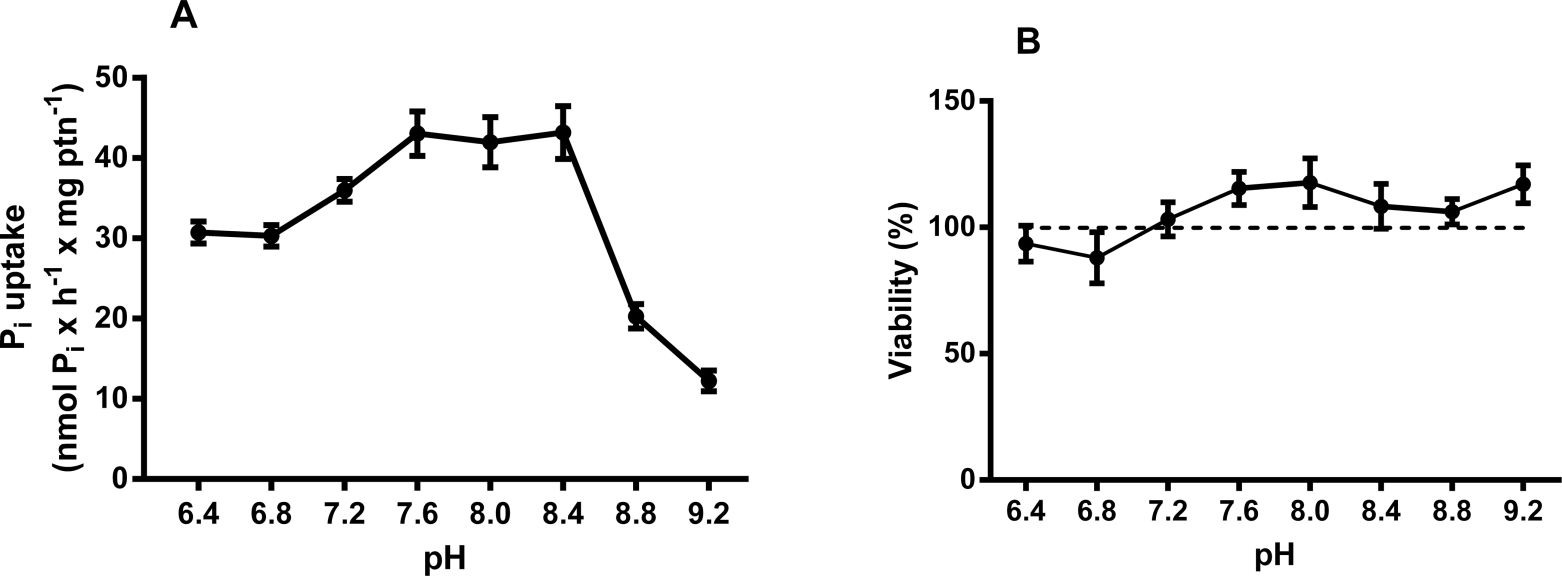
Effect of pH on NaCl-dependent ^32^Pi transport in MDA-MB-231. Intact cells (5 x 10^**4**^ cells/mL = 1.45 mg protein/mL) were incubated for 1 h at 37°C in a reaction mixture containing 116 mM NaCl or 116 mM choline cloride, 5.5 mM Glucose, 5.4 KCl, 0.8 mM MgCl_**2**_, 0.1 mM KH_**2**_PO_**4**_, 2.5 μCi/nmol ^**32**^Pi and 10 mM HEPES, 15 mM Tris, 15 mM MES with pH ranging from 6.4 to 9.2 (A). In these pH ranges, the cells remained viable throughout the experiment according CellTiter 96® AQueous One Solution Cell Proliferation Assay (MTS) (B). These results are the means ± SE of at least 3 experiments, with different cell suspensions.

pH values ranging from 6.4 to 9.2 were tested, and the transport was higher at pH 7.3–7.7, as shown in Fig 4A. Fig 4B shows the cell viability during the pH experiments. Thus, all the following experiments were performed at pH 7.2, the same as the mean pH of the cell culture medium.

**Fig 5:**
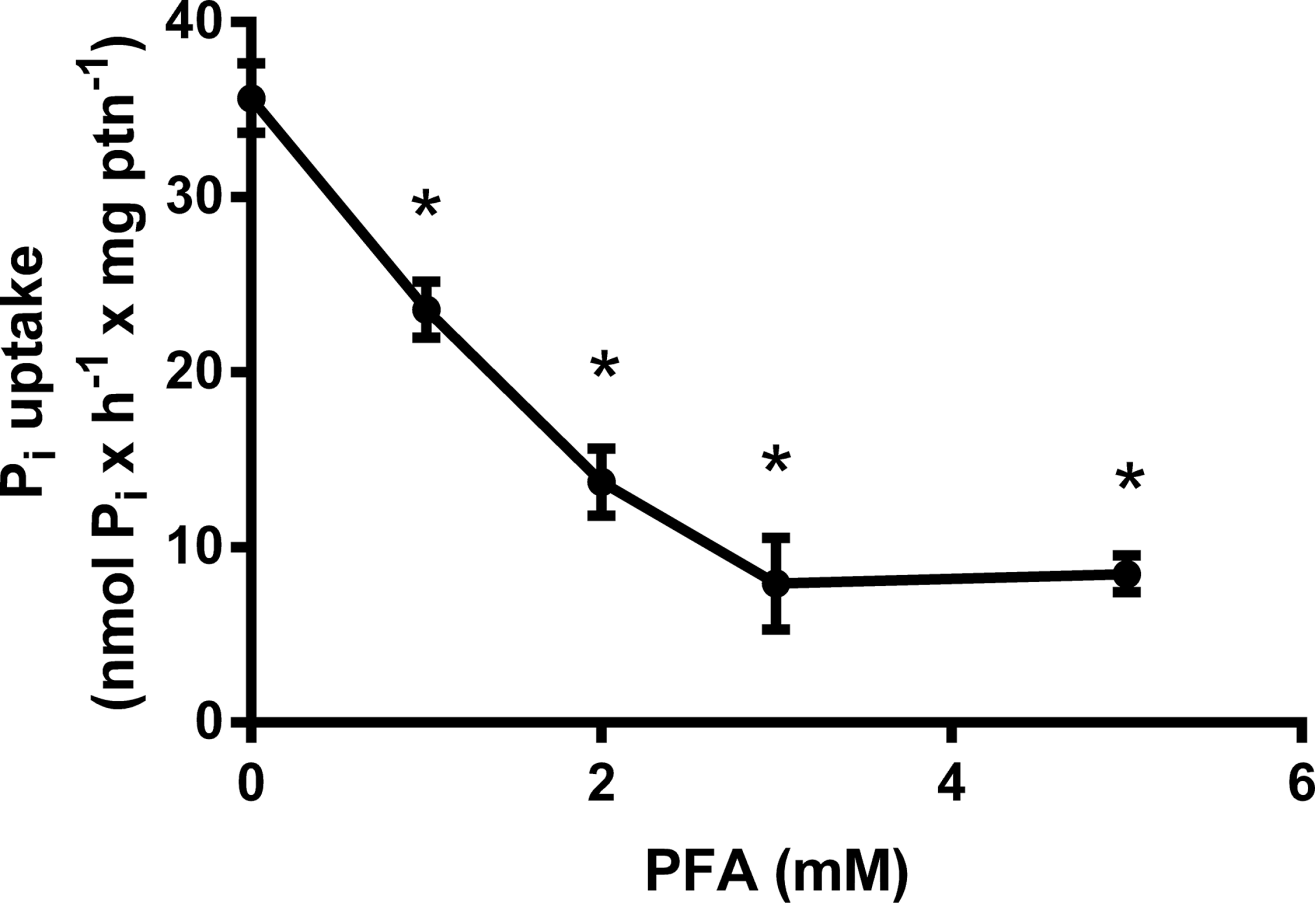
Effect of PFA on NaCl-dependent ^32^Pi transport in MDA-MB-231. Intact cells (5 x 10^4^ cells/mL = 1.45 mg protein/mL) were incubated for 1 h at 37°C in a reaction mixture containing 116 mM NaCl or 116 mM choline cloride, 5.5 mM Glucose, 5.4 KCl, 0.8 mM MgCl_2_, 0.1 mM KH_2_PO_4_, 2.5 μCi/nmol ^32^Pi and 10 mM HEPES, in the presence of increasing concentrations of PFA. In these PFA concentrations, the cells remained viable throughout the experiment. The results are the means ± SE of at least 3 experiments, with different cell suspensions. Asterisks mark significant differences (p≤ 0.05) from control, as determined by One-Way analysis of variance (ANOVA), using Turkey’s multiple comparisons test.

In order to evaluate the inhibition mechanism of Pi transport, we tested some inhibitors: bafilomycin A_1_ (100 nM), SCH28080 (100 μM), valinomycin (100 μM), ouabain (1 mM), Furosemide (1 mM), monensin (100 μM) and PFA (5 mM) (Table 2). With the purpose of verifying the influence of H^+^ gradient on the Pi uptake, we tested the vacuolar H^+^ATPase inhibitor bafilomycin A_1_ and SCH28080, a H^+^,K^+^-ATPase inhibitor, that were not able to inhibit the P_i_ transport in this cell, as well as valinomycin, a K^+^ ionophore. Only furosemide, ouabain, monensin and PFA inhibited the Pi transport. Taken together, these data suggest the importance of Na^+^ gradient to P_i_ transport, possibly by the involvement of an Na^+^,K^+^-ATPase and/or Na^+^-ATPase. The influence of PFA in P_i_ transport was also verified. Increasing concentrations of this inhibitor were tested and Fig 5 shows the dose-response to PFA. These inhibitors do not affect the viability of MDA-MB-231(Table 2).

**Table 2:** Effect of different agents on Pi transport of MDA-MB-231.

Condition	Activity (nmol P_i_ x h^-1^ x mg ptn^-1^)	Viability (% of control)
CTRL	34.61 ± 4.80	100
Bafilomycin A_1_ (100 nM)	31.57 ± 4.78	87
SCH28080 (100 μM)	34.60 ± 6.99	99
Valinomycin (100 μM)	33.70 ± 8.66	116
Ouabain (1 mM)	17.60 ± 3.67*	116
Furosemide (1 mM)	14.20 ± 3.49*	112
Monensin (100 μM)	20.70 ± 5.04*	104
PFA (5 mM)	7.65 ± 1.34*	103
Triton X 100 (1%)	-	28

Reactions were performed at 37°C in a medium (final volume: 0.5 mL) containing 116 mM NaCl or choline chloride (to calculate the Na^+^-dependent P_i_ transport), 5.4 mM KCl, 5.5 mM D-glucose, 0.8 mM MgCl_2_, 50 mM Hepes, pH 7.2, 0.1 mM KH_2_PO_4_, 2.5 μCi/nmol ^32^P_i_ and 5 x 10^4^ cells/mL (1.45 mg protein/mL) in the absence or presence of other additions, as shown in the table. The results shown are representative of at least three independent experiments. *Denotes significant differences (p < 0.05) after comparison with the control (no addition).

### Cell migration and adhesion

**Fig 6:**
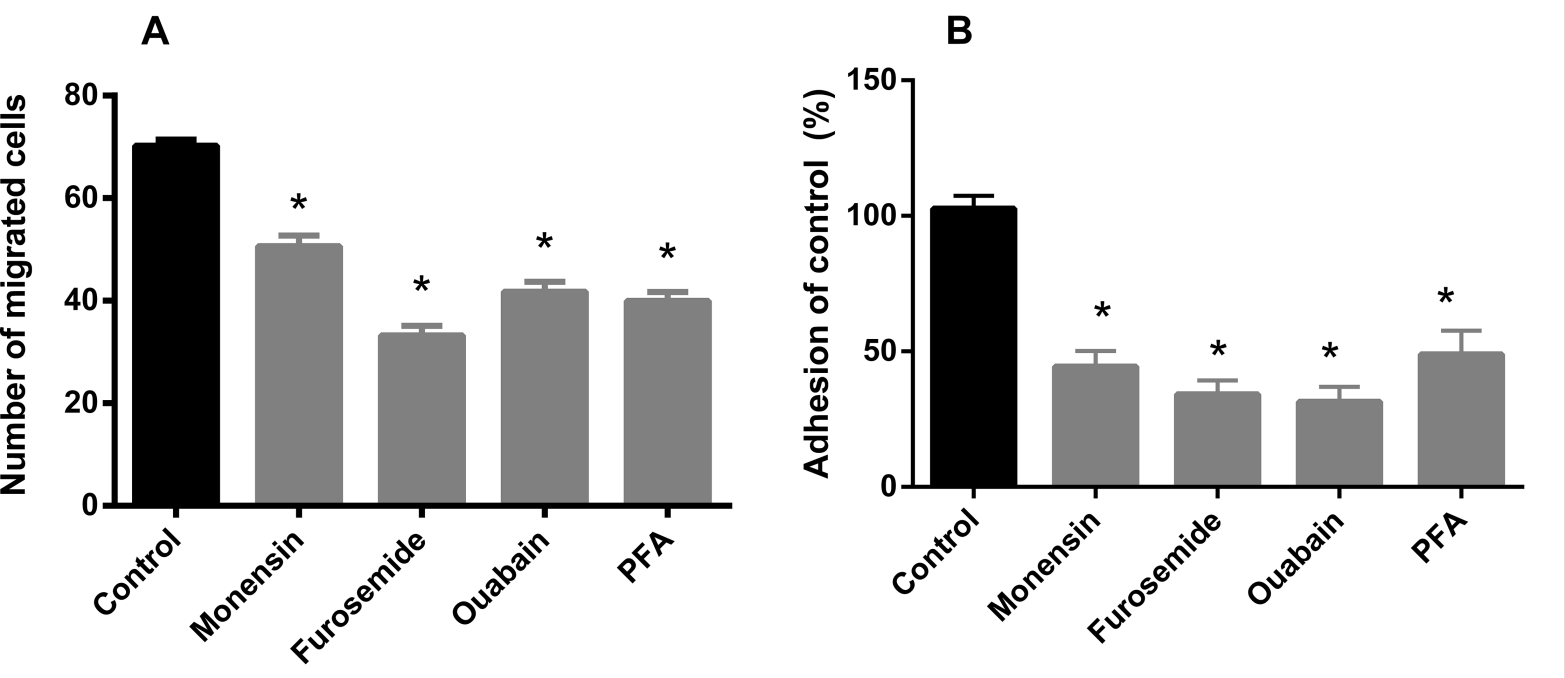
**Effect of inhibitors on cell migration (A) and cell adhesion (B) in MDA-MB-231.** Intact cells (5 x 10^5^ cells/mL) were incubated for 1 h at 37°C in a Boyden Chamber Assay™ migration (A) or in a adhesion chamber (B) in the presence or absence (control) of inhibitors indicated in the abscissa: ouabain (1 mM), furosemide (1 mM), Monensin (100 μM) and PFA (5 mM). In the presence of these inhibitors at their respective concentrations, the cells remained viable throughout the experiment. The results are the means ± SE of at least 3 experiments, with different cell suspensions. Asterisks mark significant differences (p≤ 0.05) from control, as determined by One-Way analysis of variance (ANOVA), using Turkey’s multiple comparisons test.

In order to evaluate the possible impact of Pi transport inhibition on tumor cell properties, we further tested the effect of furosemide, ouabain, monensin and PFA on cell migration and adhesion. All inhibitors were able to inhibit the migration and adhesion of MDA-MB-231 cells (Fig 6). Pi transport inhibitors decreased cell adhesion by approximately 50%. In the case of migration, the inhibition was also significant, especially in the presence of furosemide, ouabain and PFA. These results indicate that Pi uptake is important for pro-tumoral processes such as cell motility, migration and adhesion. None of the inhibitors affected cell viability (Table 2).

## Discussion

Inorganic phosphate (Pi) has been recently proposed as a key component of the “growth rate hypothesis”, in which tumors exhibit high phosphorus concentration due to the requirement of a high amount of ribosomes necessary to produce proteins that support the accelerated proliferation of cancer cells [12]. Consistent with the GRH, it was reported that some tumors have higher phosphorous and RNA content than normal tissue, and that phosphorous in RNA have an important contribution to the total biomass of phosphorous in malignant rather than normal tissues [12]. More recently, it was also demonstrated that interstitial inorganic phosphate, which is elevated in tumor microenvironment, could be a new marker of tumor progression [13]. In this study, the authors propose that measurement of interstitial inorganic phosphate would be more sensitive and specific for tumor detection, as compared to blood Pi concentration measurements [13].

In this work we evaluated the biochemical behavior of Pi transport in an aggressive breast cancer cell line, MDA-MB-231 in an attempt to understand the importance of Pi to cancer cells. Initially, we compared the Pi transport of MCF-7, T47D and MDA-MB-231 cells. The high level of Pi transport observed in MDA-MB-231 cell line may reflect aspects of the metabolism associated to the energy demands linked to its metastatic phenotype. Conceivably, the extra Pi incorporated by MDA-MB-231 cells could be utilized as a substrate in ATP biosynthesis, thus enabling cells to sustain their typical proliferation and migration patterns. Indeed, the increased expression of Pi transporters in MDA-MB-231 cells is compatible with such a view [18,30]. Other parameters have been reported that stress the distinct metabolic identities of these cell lines. For example, MCF-7, T47D and MDA-MB-231 cells differ in lactate secretion, 2-NBDG uptake, expression of LDH and sensitivity to histone deacetylase inhibitors [31]. As a matter of fact these differences should not be surprising in view of the diverse phylogenies of MCF-7, T47D and MDA-MB-231 cell lines. Regarding the expression of Pi transporters, NaPi-IIb has been shown to be highly expressed in breast cancer [23]. We observed that the NaPi-IIb had a higher expression compared to other inorganic phosphate transporters in the MDA-MB-231 cell line (Fig 2).

Because a high expression of Pi transporters has been described in breast cancer compared to normal tissue, we have sought to identify the biochemical profile of the Pi transporter (NaPi-IIb) in kinetic terms and to correlate the transport of inorganic phosphate with the tumor processes [23]. According to Forster *et al*. [6] and Takeda [4], the Pi transporter in breast cells would be sodium dependent and with high affinity for inorganic phosphate. Partially, we correlated this high affinity of the phosphate transporter in breast cancer with the high energy requirement typical of cancer cells [30].

Pi is a tryptotic acid and, thus, has different physiological forms according to the pH range in which it is found [1]. Thus, when the Pi transport levels at different pH ranges were tested, we observed higher levels of transport at neutral pH than at more alkaline pH. With this result, we verified that the sodium-dependent inorganic phosphate carrier has a higher affinity for Pi in the diprotic form (H_2_PO_4_) found in the blood (pH 7.2).

**Fig 7:**
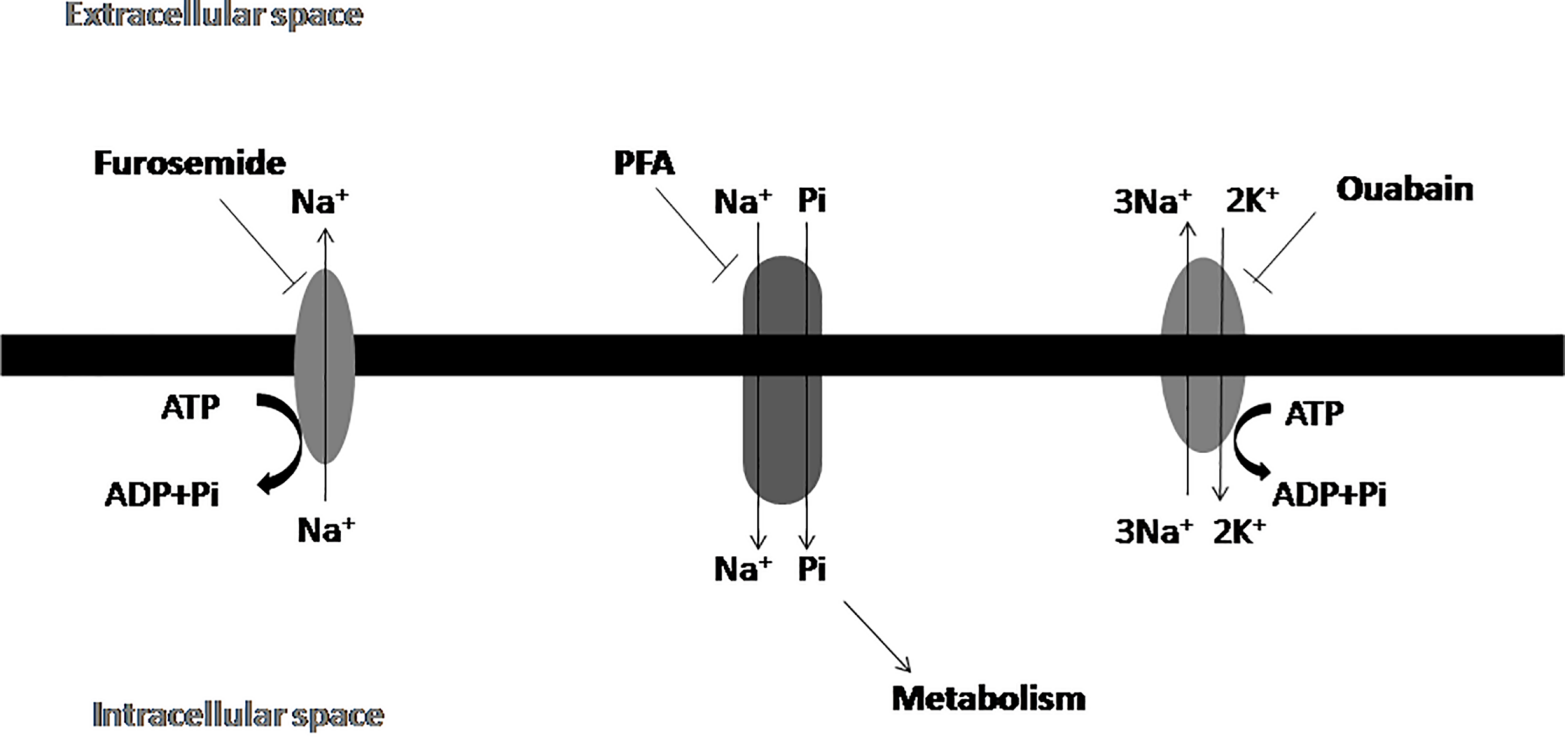
Proposed mechanism for Pi transport mechanism in MDA-MB-231 cells: Na^+^, K^+^-ATPase ouabain-sensitive in the plasma membrane and Na^+^-ATPase furosemide-sensitive plasma membrane coupled to Pi transport. Arrows indicate the direction of ion flow.

Previous studies have demonstrated that in mammary glands NaPi-IIb was prevalently expressed as the carrier of Pi, a sodium-dependent carrier capable of transporting the Pi molecule. Furthermore, we demonstrated that the Pi transporter in tumor cells was also sodium dependent. In addition, ouabain (1 mM), furosemide (1 mM) and monensin (100 μM) were able to inhibit the transport of Pi. Furosemide inhibits the Na^+^-ATPase and ouabain inhibits the Na^+^,K^+^-ATPase. As a result the Na^+^ ion accumulates in the cytosol, leading to indirect inhibition of Pi transport. Monensin is a Na^+^ ionophore, undoing the gradient of this ion across the cytoplasmic membrane. Collectively these results suggest that the inhibitors may deregulate the intracellular sodium gradient and consequently affect sodium-dependent Pi transport (Fig 7).

Phosphonoformic acid is a competitive antagonist of the Pi transporter belonging to the NaPi-II family [32]. As expected, Pi transport into breast cancer cells in the presence of this compound was inhibited in a dose dependent manner, further evidence of the existence of the Na^+^-dependent Pi transporter on the membrane of these cells.

It was recently demonstrated in other cell types [26,33,34] that some inhibitors of Pi transport, such as FCCP, valinomycin and SCH28080, could have metabolic effects at the mitochondrial level leading to severe ATP depletion, thus indirectly affecting the active Pi uptake. Therefore, we measured the intracellular ATP content under Pi transport conditions, in the absence or presence of monensin, furosemide, ouabain and PFA, which inhibit Pi transport in MDA-MB-231 cells. However, neither inhibitor modulated cellular ATP levels (S1 Fig).

Lin *et al*. [35] identified a dose-dependence of inorganic phosphate for the cell migration of the MDA-MB-231 strain, suggesting the importance of Pi for the metastatic process. Based on this result, we sought to evaluate the importance of the Pi transporter for the metastatic process. Therefore, we used the inorganic phosphate transport inhibitors (monensin, ouabain and furosemide) and the Pi transport inhibitor (PFA) in the cell migration and adhesion assays. Interestingly, we noted a significant inhibition of cell migration, as well as cell adhesion with all inhibitors of Pi transport. PFA has been reported to inhibit the NaPi transport in osteoclast cells [36]. From these results it can be inferred that Pi directly (free Pi), or indirectly (conjugated as ATP) could be accessory to the glycolytic pathway and thus play a role in supplying energy for cellular motility and adhesion of breast cancer cells. Pi uptake appears to be related to the sodium gradient rather than to the Pi transporter itself since ouabain, furosemide and monensin decreased Pi transport, cell migration and adhesion. Therefore, the use of phosphonoformic acid clearly demonstrated that the cells were fully dependent on Pi transport for such functions.

In this study, we characterized the Pi transport in the triple-negative breast cancer cell line, MDA-MB-231. The Pi transport was found to be Na^+^-dependent, had high affinity for Pi and was more active in acid pH range; it was also inhibited by classical Pi transport inhibitor, PFA. Remarkably, the inhibition of Pi transport caused significant decrease in tumor cell migration and adhesion, suggesting a prominent role for Pi transporters in tumor progression. In this context, these proteins might be regarded as potential therapeutic targets in breast cancer.

## Supporting information

S1 Fig Effect of inhibitors on ATP levels MDA-MB-231. Intact cells were incubated for 1h at 37°C in a reaction mixture containing 116 mM NaCl or 116 mM choline cloride, 5.5 mM Glucose, 5.4 KCl, 0.8 mM MgCl2 and 10 mM HEPES, in the presence of the indicated inhibitors: monensin, furosemide, ouabain or PFA, as shown in the abscissa; 0.1 mM KH2PO4 to measure the ATP levels using an ATP bioluminescent assay Kit. The data shown are the mean activities ± SE of at least three determinations, each with different cell suspensions. Asterisks mark significant differences (p < 0.05) from control One-Way analysis of variance (ANOVA), using Turkey’s multiple comparisons test. CTRL: control of the experiment of Pi transport in the presence of inhibitors vehicles (water, ethanol or dimethyl sulfoxide).
(TIF)

## Abbreviations

DMSO: dimethyl sulfoxide
EDTA: ethylenediamine tetraacetic acid
FBS: foetal bovine serum
HEPES: 4-(2-hydroxyethyl)-1-piperazineethanesulfonic acid, PFA, phosphonoformic acid
